# AKT and 14-3-3 Regulate Notch4 Nuclear Localization

**DOI:** 10.1038/srep08782

**Published:** 2015-03-05

**Authors:** Gopalakrishnan Ramakrishnan, Gantulga Davaakhuu, Wen Cheng Chung, He Zhu, Ajay Rana, Aleksandra Filipovic, Andrew R. Green, Azeddine Atfi, Antonio Pannuti, Lucio Miele, Guri Tzivion

**Affiliations:** 1 Cancer Institute, University of Mississippi Medical Center, Jackson, MS 39216; 2 Department of Molecular Pharmacology and Therapeutics, Loyola University Chicago, Maywood, IL 60153; 3 Imperial College London, Division of Surgery and Cancer, Department of Oncology, Hammersmith Hospital Campus, Du Cane Road, London, W12 0NN, UK; 4 Department of Histopathology and School of Molecular Medical Sciences, University of Nottingham, Nottingham City Hospital, Nottingham, NG5 1PB, UK; 5 Department of Biochemistry, University of Mississippi Medical Center, Jackson, MS 39216

## Abstract

Members of the Notch family of transmembrane receptors, Notch1-4 in mammals, are involved in the regulation of cell fate decisions and cell proliferation in various organisms. The Notch4 isoform, which is specific to mammals, was originally identified as a viral oncogene in mice, Int3, able to initiate mammary tumors. In humans, Notch4 expression appears to be associated with breast cancer stem cells and endocrine resistance. Following ligand binding, the Notch4 receptor undergoes cleavage at the membrane and the Notch4-intracellular domain (ICD), translocates to the nucleus and regulates gene transcription. Little is known on the mechanisms regulating Notch4-ICD and its nuclear localization. Here, we describe the identification of four distinct AKT phosphorylation sites in human Notch4-ICD and demonstrate that AKT binds Notch4-ICD and phosphorylates all four sites *in vitro* and *in vivo*. The phosphorylation in cells is regulated by growth factors and is sensitive to phosphatidyl inositol-3 kinase (PI3K) inhibitors. This phosphorylation generates binding sites to the 14-3-3 regulatory proteins, which are involved in the regulation of nucleocytoplasmic shuttling of target proteins, restricting phosphorylated Notch4-ICD to the cytoplasm. Our findings provide a novel mechanism for Notch4-ICD regulation, suggesting a negative regulatory role for the PI3K-AKT pathway in Notch4 nuclear signaling.

The evolutionarily conserved Notch family of transmembrane receptors are involved in the regulation of cell fate decisions and cell proliferation via their activity as transcriptional regulators^1^. Canonical Notch signaling is mediated by the transcription factor CSL (CBF-1, Suppressor of Hairless, Lag-1), also known as RBP-Jκ. Mammals express four Notch genes, Notch1-4, with distinct functions and regulation, though there is some functional redundancy^2^,^3^. Notch4, which is specific to mammals, was initially identified as a truncated oncogenic form in mice, Int3, able of initiating mammary tumors^4^,^5^. The oncogenic activity of Notch4-ICD in transgenic mice has been shown to be independent of CSL, and thus presumably requires a yet to be identified non-canonical signaling pathway^6^. In humans, Notch4 overexpression is associated with breast cancer stem cells and with endocrine resistance in estrogen-receptor α (ERα)-positive breast cancer^7^,^8^. In normal development, Notch4 expression appears to be restricted to endothelial cells^9^,^10^,^11^, where it regulates vascularization during development, as well as endothelial cell function and response to inflammation^11^,^12^,^13^,^14^. It has also a role in tumor angiogenesis and blood vessel perfusion^15^,^16^.

Activation of Notch receptors is initiated by the binding of transmembrane ligands on adjoining cells, such as Delta and Serrate/Jagged families to the Notch receptor, leading to its sequential cleavage and the release of the intracellular domain, designated Notch-ICD^17^. This fragment translocates to the nucleus and participates in the formation of specific transcriptional complexes^1^,^18^,^19^. Much of what is known about Notch signaling derives from studies of Notch1 and Notch2, the most conserved paralogs. Non-canonical, non-nuclear signaling has been described for Notch1 in T-cells. Specifically, Notch1 has been reported to signal through AKT via mTORC2 and to NF-κB via the IKK signalosome and nuclear IKKα^20^,^21^,^22^,^23^. Notch-ICD is a relatively short-lived protein and undergoes rapid degradation through an ubiquitin-mediated pathway. The pathways that regulate the stability of Notch-ICDs other than Notch1 as well as the protein complexes they form are still poorly defined. Specifically, the regulation of Notch4-ICD by phosphorylation or other post-translational modifications or by interaction with adapter proteins, which are key regulators of many other transcription modulators, is largely unknown^24^. A recent study demonstrated direct interaction of p53 and Mdm2 with Notch4-ICD, leading to Notch-4 degradation by an ubiquitin-mediated pathway^25^. Conversely, Mdm2 has been reported to activate Notch1-ICD^26^. Elongin C complexes were also shown to regulate Notch4-ICD degradation in connection with diabetic kidney fibrosis^27^. Several signaling pathways, including the PI3K-AKT pathway, have been shown to crosstalk with Notch family members (Notch1 and 3), however, the mechanistic aspects of this crosstalk have not been defined^28^,^29^.

Here, we describe the identification of four distinct AKT phosphorylation sites in human Notch4-ICD and demonstrate that these sites are phosphorylated by AKT *in vitro* and *in vivo*. In turn, these phosphorylations generate binding sites for the regulatory 14-3-3 scaffold proteins. The Notch4-ICD-14-3-3 binding in cells is regulated by growth factors and is dependent on the PI3K-AKT pathway. This regulatory network controls the nuclear localization of Notch4-ICD by excluding the phosphorylated Notch4-ICD from the nucleus and thus potentially restricting its transcriptional activity. The identified regulatory mechanism is specific for Notch4 since the other Notch family members do not contain any AKT phosphorylation sites. Our results detail a new regulatory mechanism specific for controlling Notch4 ICD localization, defining a potential regulation of Notch4 function by growth factors and other signals that activate AKT.

## Results

### Notch4-ICD contains AKT consensus phosphorylation sites and binds AKT

**Figure 1:**
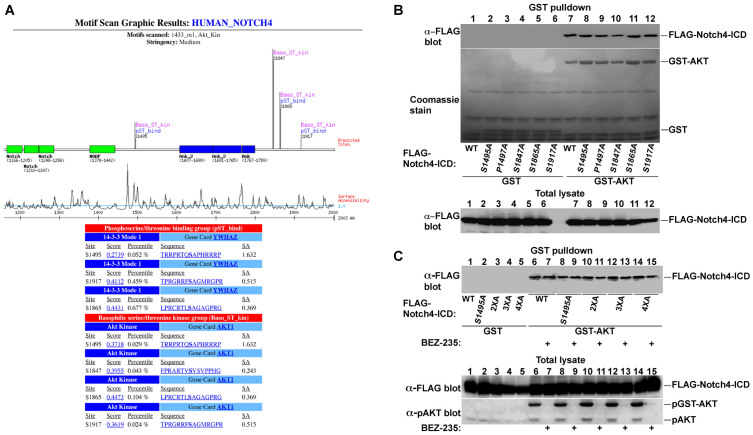
Human Notch4-ICD contains four putative AKT phosphorylation sites and binds AKT *in vivo*. (A) The human Notch4 protein sequence (accession # AAC32288) was analyzed using the Motif Scan program. Presented are the results of the analysis for AKT phosphorylation and 14-3-3 binding sites in the ICD domain. Note the overlap between the 14-3-3 binding sites and the AKT phosphorylation sites. (B & C) COS-7 cells were transfected with GST control or GST-AKT together with FLAG-Notch4-ICD or its mutants as indicated. Exponentially growing cells (B) or cells treated with vehicle or 10 nM of the PI3K inhibitor BEZ235 (C) were analyzed for FLAG-Notch4-ICD recovery in GST pull-downs by FLAG immunoblotting. GST and GST-AKT protein recoveries were analyzed by coomassie staining (B). Notch4-ICD expression and AKT phosphorylation (S473) were analyzed in total cell lysates. WT: wildtype; 2XA: S1495/1847A; 3XA: S1495/1847/1865A; 4XA: S1495/1847/1865/1917A.

The four mammalian Notch receptors show significant variations in their extracellular and intracellular domains^2^. To explore potential differences in their regulation by phosphorylation, we analyzed the four mammalian Notch receptors for the presence of phosphorylation motifs using the Motif Scan program (http://scansite.mit.edu)^30^ that predicts protein phosphorylation sites and protein interaction motifs. This analysis revealed the presence of four consensus AKT phosphorylation sites in human Notch4-ICD: S1495, S1847, S1865 and S1917 (Fig 1A). These sites are not conserved in the other Notch receptors, suggesting a specific mechanism for Notch4 regulation by AKT. Interestingly, these four sites are fully conserved in primates, but are only partially conserved in other mammals, including rodents and larger mammals (Supplementary Fig 1 and data not shown), pointing to a somewhat distinctive mechanism for the regulation of human and primate Notch4 proteins.

To determine the physiological relevance of the identified sites, we examined the interaction of AKT and Notch4-ICD *in vivo* using co-transfection experiments and GST-pull-downs (Fig 1B and C). We found extensive Notch4-ICD co-purification with GST-AKT but not with GST control (Fig 1B, compare lanes 1–6 with 7–12), demonstrating specific binding and indicating on physical interaction between AKT and Notch4-ICD. Interestingly, in contrast to several other AKT targets, such as the FoxO transcription factors that require AKT activation for binding^31^,^32^, AKT binding to Notch4 was not dependent on AKT activity (Fig 1C, BEZ-235 treatment) or the presence of the AKT phosphorylation sites on Notch4 (Fig 1B, compare lane 7 with 8–12 and Fig 1C, lanes 6,7 with 8–15), suggesting that AKT constitutively binds to Notch4-ICD.

### AKT phosphorylates Notch4-ICD *in vitro* and *in vivo*

**Figure 2:**
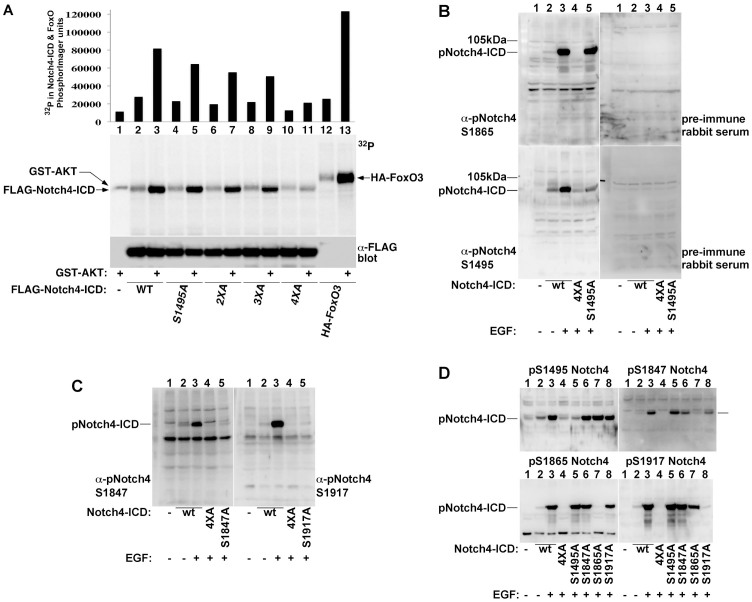
AKT phosphorylates Notch4-ICD on four distinct sites *in vitro* and *in vivo*. (A) The indicated FLAG-Notch4-ICD forms and HA-FoxO3 were purified by immunoprecipitation from HEK-293T cells and incubated alone or with active AKT in a kinase reaction mixture.^32^P incorporation in Notch4-ICD and FoxO3 were quantified by phospho-imaging (top panel). Notch4-ICD recovery was visualized by FLAG immunoblotting. (B–D) Protein extracts from COS-7 cells expressing the indicated FLAG-Notch4-ICD forms, treated with vehicle or EGF (100 ng/ml for 15 min), were immunoblotted with the indicated phospho-Notch4 antibodies or pre-immune rabbit serum (B). Indicated are the migration points of Notch4-ICD and the 105 kDa protein marker (B).

To determine whether AKT can directly phosphorylate Notch4-ICD, we performed *in vitro* kinase assays using purified AKT and either wildtype Notch4-ICD or mutant forms substituted with alanines at the predicted AKT phosphorylation sites (Fig 2A). These experiments showed that AKT can directly phosphorylate Notch4-ICD and that all four identified sites contribute to the phosphorylation *in vitro*. AKT did not phosphorylate a mutant form lacking all four sites (Fig 2A, compare lanes 3 and 11), indicating that the identified sites are the only Notch4-ICD sites phosphorylated by AKT *in vitro*.

In order to study the regulation of Notch4-ICD phosphorylation on the identified sites *in vivo*, we developed phosphospecific antibodies for each of the phosphorylation sites (Fig 2B, C and D). We were successful in obtaining site-specific antibodies with minimal cross-reaction with unphosphorylated Notch4 or among the four AKT phosphorylation sites (Fig 2B, C and D). Using these antibodies, we found that all the four predicted Notch4-ICD sites are phosphorylated *in vivo*. Importantly, reactivity with these antibodies required cell stimulation with growth factors (e.g., EGF) pointing to the dependency of the phosphorylation on the PI3K-AKT pathway (Fig 2 B, C and D, compare lanes 2 and 3).

### AKT phosphorylation promotes Notch4-ICD interaction with 14-3-3ζ

**Figure 3:**
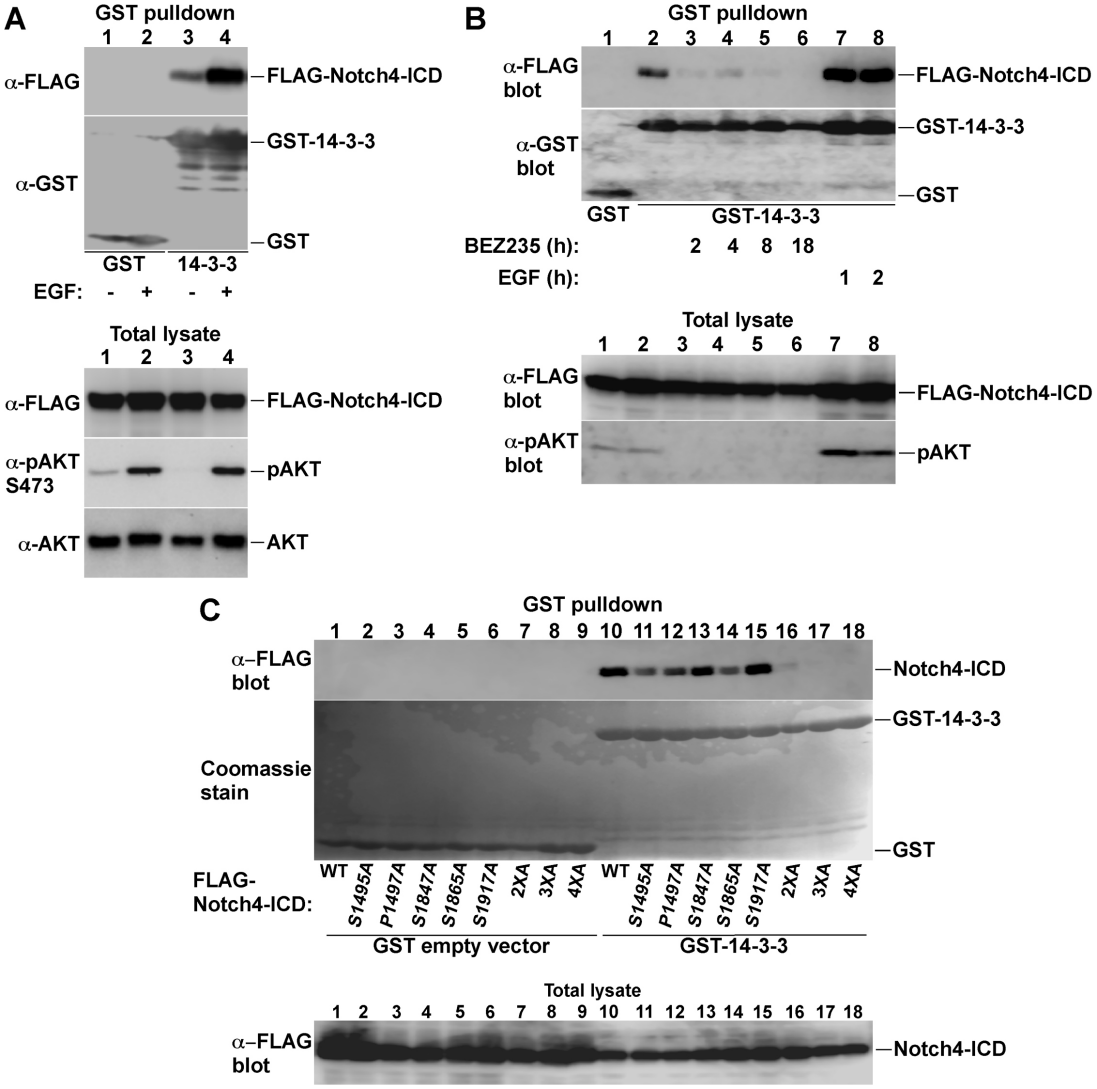
AKT phosphorylation promotes Notch4-ICD binding to 14-3-3ζ. (A & B) COS-7 cells were transfected with GST control or GST-14-3-3ζ together with FLAG-Notch4-ICD as indicated. Cells were treated with vehicle or 100 ng/ml EGF for 15 min (A) or for the indicated periods (B) or with 10 nM of BEZ235 for the indicated periods (B). Samples were analyzed for FLAG-Notch4-ICD recovery in GST pull-downs by FLAG immunoblotting. GST and GST-14-3-3ζ protein recoveries were analyzed by GST immunoblotting. Notch4-ICD expression and AKT phosphorylation were analyzed in total cell lysates. (C) The indicated FLAG-Notch4-ICD forms were coexpressed with GST or GST-14-3-3ζ in HEK-293T cells and FLAG-Notch4-ICD recovery in GST pull-downs was examined as in A & B. Note that HEK293T cells have constitutive activation of AKT, allowing constitutive GST-14-3-3-Notch4-ICD interaction.

AKT phosphorylation of target proteins often generates binding sites for the 14-3-3 adaptor protein family, and in many cases, 14-3-3 binding rather than the phosphorylation itself is responsible for regulating the function of the target protein^32^,^33^,^34^,^35^. Indeed, the Motif Scan analysis identified several consensus 14-3-3 binding sites in Notch4-ICD, overlapping with the AKT phosphorylation sites at S1495, S1865 and S1917 (Fig 1A). The site surrounding S1495 showed the strongest 14-3-3 binding consensus from all three sites, suggesting it could serve as the primary 14-3-3 binding site. To examine whether 14-3-3 binds to Notch4-ICD, we used similar GST-pull-down experiments as with AKT, demonstrating *in vivo* interaction between 14-3-3ζ and Notch4-ICD (Fig 3A). The binding was induced by growth factor treatment and was sensitive to PI3K inhibition (Fig 3A and B), indicating regulation through the PI3K-AKT pathway. The binding was also dependent on the AKT phosphorylation sites, as individual mutation of the AKT phosphorylation sites decreased the binding to 14-3-3 and mutation of all four sites completely eliminated the binding (Fig 3C). The results also showed that the site surrounding S1495 is the main binding site, since mutation of this site resulted in the most marked reduction in 14-3-3 binding compared to all other single mutations (Fig 3C, compares lanes 10 and 11 with 13–15). Supporting this notion, mutation of proline 1497 diminished 14-3-3 binding (Fig 3C, lane 12), consistent with the dependency of 14-3-3 proteins on proline at the +2 position of the phosphorylated residue for optimal binding^31^,^36^. 14-3-3 proteins bind their targets as dimers. The binding is facilitated by the presence of two or more binding sites on the target proteins and usually depends on one optimal site for the initial interaction, which then facilitates the binding of the other chain to less optimal sites^34^,^36^. Our results suggest that the site at S1495 serves as this initial interaction site and that the other, less optimal sites, serve as the secondary interaction points. Interestingly, this site is only present in primates but is not conserved in other mammals (Supplementary Fig 1 and data not shown), suggesting that the 14-3-3-mediated regulation of Notch4-ICD is somewhat unique to primates.

### AKT-phosphorylated Notch4-ICD is restricted to the cytoplasm

**Figure 4:**
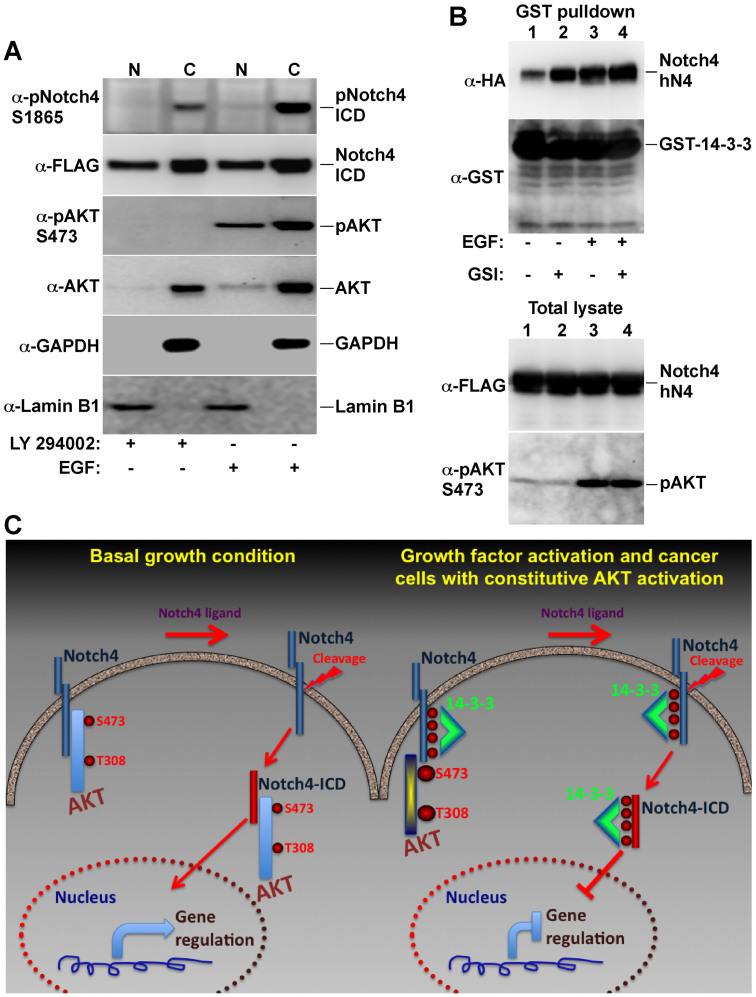
AKT-phosphorylated Notch4-ICD is restricted to the cytoplasm. (A) COS-7 cells expressing FLAG-Notch4-ICD were treated with 10 μM of the PI3K inhibitor LY 294002 for 1 h or with 100 ng/ml EGF for 15 min and cells were fractionated for nuclear and cytoplasmic fractions. Equal amount of nuclear and cytoplasmic protein was analyzed by immunoblotting using the indicated antibodies. Note the exclusion of phosphorylated-Notch4-ICD from the nuclear fraction (top panel). (B) 14-3-3-Notch4 association was analyzed in COS-7 cells co-expressing GST-14-3-3ζ and HA-Notch4-ΔhN4 (Notch4 form that contains the transmembrane region and parts of the extracellular domain and requires proteolytic cleavage for releasing the ICD domain). Cells were treated as indicated with the γ-secretase inhibitor BMS-708163 (GSI, 1 μM for 1 h), EGF (100 ng/ml for 15 min) or their combination. Note that the GSI treatment increased Notch4-14-3-3 association without affecting the AKT activation level. (C) A model for Notch4-ICD regulation by AKT and 14-3-3. Based on our results, we propose that AKT is constitutively associated with Notch4 at the membrane in basal conditions. In this situation, activation of Notch4 by one of its ligands will result in the release of active Notch4-ICD that will translocate to the nucleus and participate in gene regulation. Whether AKT is bound to Notch4-ICD in the nucleus remains to be determined. In growth factor-activated cells or cancer cells with constitutive AKT activation, Notch4-ICD is phosphorylated by AKT and is bound to 14-3-3 at the membrane. Notch4 receptor activation results in release of Notch4-ICD, however, 14-3-3 restricts its translocation to the nucleus and participation in gene regulation.

14-3-3 binding can regulate the function of its targets by several mechanisms: it can affect stability and dephosphorylation, increase or decrease enzymatic activity and alter subcellular localization^33^. In the case of FoxO transcription factors, for example, 14-3-3 proteins have been shown to exert all three functions: restricting their nuclear localization, obstructing their binding to DNA and stabilizing the phosphorylated form^31^,^32^,^37^. To determine the effects of AKT phosphorylation and the concomitant 14-3-3 binding on Notch4-ICD localization, we fractionated cells into cytoplasmic and nuclear fractions and examined the presence of total and phosphorylated Notch4-ICD in the fractions (Fig 4A). These experiments demonstrated that the phosphorylated Notch4-ICD was present only in the cytoplasm, while total Notch4-ICD was both in the cytoplasm and the nucleus (Fig 4A). These results were similar to our results with FoxO transcription factors, suggesting that the AKT-14-3-3 network acts to restrict the translocation of Notch4-ICD to the nucleus. To examine whether 14-3-3 binding to the Notch4-ICD occurs before or after cleavage of the receptor, we examined the binding of 14-3-3 to Notch4-ΔhN4, which contains the transmembrane domain and is anchored in the membrane (Fig 4B). We obtained a robust binding of 14-3-3 to this Notch4 form and the binding was stabilized by treating the cells with γ-secretase inhibitors that block cleavage of the Notch4-ICD domain (Fig 4B, compare lanes 1 and 2). The results suggest that Notch4 phosphorylation by AKT and the binding of 14-3-3 occur already at the membrane before the cleavage of the ICD domain.

## Discussion

The results presented in this study suggest a novel mechanism for the posttranslational regulation of Notch4-ICD (Fig 4C). This regulation involves the binding of inactive AKT to Notch4, possibly at the membrane, and phosphorylation of Notch4-ICD upon AKT activation. This phosphorylation in turn generates binding sites for the regulatory 14-3-3 proteins. Our data suggests that the Notch4-ICD could be pre-bound to 14-3-3 before cleavage and that 14-3-3 binding restricts the phosphorylated Notch4-ICD to the cytoplasm. It remains to be determined whether 14-3-3 binding also restricts the incorporation of Notch4-ICD into active transcriptional complexes or its association with chromatin by masking the RAM domains on Notch4-ICD and whether it has a role in controlling the Notch4-ICD half-life by affecting its degradation. The physiological function of phosphorylated, 14-3-3-bound Notch4-ICD remains to be clarified. It may serve as a reservoir to be mobilized after another signal triggers its dephosphorylation, and/or it may signal through non-canonical, cytoplasmic pathways.

Our findings have potentially strong implications to understanding normal developmental processes that involve Notch4 function as well as cancer pathogenesis by providing a novel link between insulin and growth factor-activated AKT signaling and the transcriptional regulator Notch4. AKT is activated in 50% of breast and prostate cancers and promotes tumorigenesis by teaming with 14-3-3 to regulate key proteins involved in cellular transformation, such as FoxO transcription factors, the pro-apoptotic protein BAD and the cell cycle regulator p27Kip1 among others. To explore whether this crosstalk may operate in human breast cancer, we examined the well-characterized Nottingham-Tenovus breast cancer cohort TMA (n = 1079) by immunohistochemistry for pAKT (S473) and Notch4 (unpublished observation). Interestingly, pAKT correlated inversely with high nuclear Notch4 (Pearson Chi square p = 0.023) and directly with cytoplasmic Notch4 (p = 0.036). This suggests that the mechanism we have uncovered may operate in breast cancer. Notch inhibitors, as well as PI3K and AKT inhibitors, are in clinical trials, but their optimal use, as for other targeted drugs, will require mechanism-based combinations. Though there is strong evidence that Notch receptors cooperate with the PI3K-AKT pathway in breast cancer, the mechanism of this crosstalk and thus the potential therapeutic implications of combining their inhibitors is not well defined. Our data suggests that AKT inhibition may result in release of Notch4-ICD from 14-3-3 and increased nuclear Notch4 signaling. The possible role of this pathway in the non-canonical oncogenic activity of Notch4-ICD remains to be determined. Interestingly, mammary mouse tumors caused by expression of Notch4-ICD display high levels of pAKT and pMEK, and cell lines derived from these tumors require PI3K and ERK activities for anchorage-independent growth^38^. However, whether murine Notch4 is also regulated by AKT and/or 14-3-3 through partially conserved sites (Supplementary Fig 1) remains to be determined.

Based on our data, it is also possible that Notch4-ICD may act as an AKT scaffold, facilitating AKT signaling and potentially transporting AKT into the nucleus. Evidence also exists that Notch4 is essential for the maintenance of the elusive breast cancer stem cells. Thus, the identification of Notch4 as a direct AKT target could provide a breakthrough in understanding Notch4 regulation and highlight a rationale for combining Notch4 and AKT targeting drugs.

## Methods

### cDNA constructs, antibodies and inhibitors

pEBG-GST-AKT, pEBG-GST-14-3-3ζ and pcDNA3.1-HA-FoxO3 were as previously described^31^. pCDNA3-FLAG-Notch4-ICD and pCDNA3-HA-Notch4-ΔhN4 were gifts from Brian Nickoloff (present address: Michigan State University Medical School) and Keith Brennan (University of Manchester). The QuikChange site-directed and the multi-site-directed mutagenesis kits (Stratagene) were used to generate the Notch4-ICD mutants: S1495A, P1497A, S1847A, S1865A, S1917A and their combinations: S1495/1847A (2XA), S1495/1847/1865A (3XA), S1495/1847/1865/1917A (4XA) and were confirmed by full-length sequencing. Pan-AKT, p-S473-AKT, GST, GAPDH and Lamin B1 antibodies were from Cell Signaling Technology. FLAG was from Sigma. HA antibody was produced using the 12CA5 hybridoma. Phosphospecific antibodies for Notch4-S1495, S1847, S1865 and S1917 were generated by Twentyfirst Century Biochemicals (Marlboro, MA). LY294002, BEZ235 and BMS-708163 were from Selleck Chemicals.

### Cell culture and transfection

HEK-293T and COS-7 cells were maintained in high-glucose DMEM media (Invitrogen), supplemented with 10% Fetal Bovine Serum in 5% CO_2_. Transient transfections were performed using FuGENE HD (Roche) according to the manufacturer's instructions.

### Cell extraction, fractionation and western blot analysis

Cellular protein was extracted in lysis buffer containing 50 mM Tris-Cl, pH 7.5, 100 mM NaCl, 1% Triton, 1 mM EDTA, 1 mM EGTA, 1 mM DTT, 50 mM β-glycerolphosphate, 2 mM Na_2_VO_4_ and protease inhibitors and cleared by centrifugation. The NE-PER fractionation kit (Pierce) was used for nuclear/cytoplasmic fractionation according to the manufacturer's instructions. Protein extracts were separated using SDS-PAGE, transferred to 0.45 uM Immobilon-P PVDF membrane (Millipore) and immuno-blotted using the indicated antibodies followed by ECL. The blots were analyzed using ChemiDoc digital imaging system (BioRad).

### Immunoprecipitation and protein binding assays

Cells expressing the indicated vectors were lysed and proteins were extracted as detailed above. Equal amounts of protein (1–2 mg/point) were incubated with protein A/G agarose beads (Santa Cruz Biotechnology) coupled with the appropriate antibody or with GSH sepharose beads (Amersham/GE Healthcare) for GST purification for 90 minutes followed by 2X washes with lysis buffer, 1X wash with lysis buffer containing 0.5 M LiCl and 2X washes with buffer containing 40 mM Tris-Cl, pH 7.5, 0.1 mM EDTA and 5 mM MgCl2. For the last wash, the beads were transferred to a new tube and the proteins were eluted using SDS-PAGE sample buffer at 95°C for 5 minutes. Total cell extracts from each sample were saved to examine protein expression and pathway activation/inhibition.

### *In vitro* kinase assay

HA-FoxO3, FLAG-Notch4-ICD and the indicated Notch4 mutants were purified by HA or FLAG-immunoprecipitation from HEK-293T cells expressing the corresponding vectors and the agarose beads were incubated with 200 ng active GST-AKT that was affinity-purified from HEK-293T cells using GSH purification. The kinase assays were performed in a kinase buffer containing 40 mM Tris-HCl, pH 7.5, 0.1 mM EDTA, 5 mM MgCl2, 2 mM dithiothreitol and 100 μM ATP supplemented with [γ-32P]ATP (10 μCi/reaction) at 30°C for 20 min. HA-FoxO3 served in the experiments as a positive AKT substrate control.

## Author Contributions

G.R. and G.D. conceived the project, designed and preformed the experiments. W.C.C. participated in the kinase experiments. A.R., A.A. and A.P. contributed to project design, data interpretation, discussions and writing. H.Z., A.F. and A.R.G. preformed the Notch4 and pAKT expression analysis in the breast cancer samples. G.T. and L.M. conceived and directed the project and wrote the manuscript.

## Supplementary Material

Supplementary Information Ramakrishnan, Supplementary Material
