# Supplementary material for: AKT and 14-3-3 Regulate Notch4 Nuclear Localization
Source: Sci Rep. 2015 Mar 5;5:8782. doi: 10.1038/srep08782 (PMC4350099; doi:10.1038/srep08782)
Supplement: Supplementary Information — Ramakrishnan, Supplementary Material [file srep08782-s1.pdf]

# Supplementary Material

## AKT and 14-3-3 Regulate Notch4 Nuclear Localization

Gopalakrishnan Ramakrishnan[1], Gantulga Davaakhuu[1], Wen Cheng Chung[1], He Zhu[1], Ajay Rana[2], Aleksandra Filipovic[3], Andrew R. Green[4], Azeddine Atfi[1], Antonio Pannuti[1], Lucio Miele[1*] and Guri Tzivion[1*]

## Figure Legends

**Supplementary Figure 1. Partial conservation of the AKT phosphorylation and 14-3-3 binding sites between human and mouse Notch4.** Alignment of human (AAC32288) and mouse (AAC52630) Notch4 proteins. Indicated are the start point of mouse Int3, the proteolytic cleavage sites (S3 and S4) and the pest domain. Note that the serine at position 1495 in human Notch4 is glutamine in mouse Notch4, eliminating the main 14-3-3 binding site. Also, the serine at position 1847 is human Notch4 is proline in the mouse. The phosphorylation sites at Ser 1865 and 1917 are conserved. The mouse has one putative new AKT phosphorylation site close to the human S1917 site (highlighted in cyan).

```
AAC32288   1281   GGDCRPEDGDPEWGPSLALLVVLSPPALDQQLFALARVLSLTLRVGLWVRKDRDGRDMVYPYPGARAEEKLGGTRDPTYQ   1360
AAC52630   1277   GGDCRPEGEDSEGRPSLALLVVLRPPALDQQLLALARVLSLTLRVGLWVRKDSEGRNMVFPYPGTRAKEELSGARDSSSW   1356

                                                                          Int3            S3
AAC32288   1361   ERAAPQTQPLGKETDSLSAGFVVVMGVDLSRCGPDHPASRCPWDPGLLLRFLAAMAAVGALEPLLPGPLLAVHPHAGTAP   1440
AAC52630   1357   ERQAPPTQPLGKETESLGAGFVVVMGVDLSRCGPEHPASRCPWDSGLLLRFLAAMAAVGALEPLLPGPLLAAHPQAGTRP   1436

                       Transmembrane     S4                          1495
AAC32288   1441   PANQLPWPVLCSPVAGVILLALGALLVLQLIRRRRREHGALWLPPGFTRRPRTQSAPHRRRPPLGEDSIGLKALKPKAEV   1520
AAC52630   1437   PANQLPWPILCSPVVGVLLLALGALLVLQLIRRRRREHGALWLPPGFIRRPQTQQAPHRRRPPLGEDNIGLKALKPEAEV   1516

AAC32288   1521   DEDGVVMCSGPEEGEEVGQAEETGPPSTCQLWSLSGGCGALPQAAMLTPPQESEMEAPDLDTRGPDGVTPLMSAVCCGEV   1600
AAC52630   1517   DEDGVAMCSGPEEGE----AEETASASRCQLWPLNSGCGELPQAAMLTPPQECESEVLDVDTCGPDGVTPLMSAVFCGGV   1592

AAC32288   1601   QSGTF---QGAWLGCPEPWEPLLDGGACPQAHTVGTGETPLHLAARFSRPTAARRLLEAGANPNQPDRAGRTPLHAAVAA   1677
AAC52630   1593   QSTTGASPQRLGLGNLEPWEPLLDRGACPQAHTVGTGETPLHLAARFSRPTAARRLLEAGANPNQPDRAGRTPLHTAVAA   1672

AAC32288   1678   DAREVCQLLLRSRQTAVDARTEDGTTPLMLAARLAVEDLVEELIAAQADVGARDKWGKTALHWAAAVNNARAARSLLQAG   1757
AAC52630   1673   DAREVCQLLLASRQTTVDARTEDGTTPLMLAARLAVEDLVEELIAARADVGARDKRGKTALHWAAAVNNARAARSLLQAG   1752

AAC32288   1758   ADKDAQDNREQTPLFLAAREGAVEVAQLLLGLGAARELRDQAGLAPADVAHQRNHWDLLTLLEGAGPPEARHKATPGREA   1837
AAC52630   1753   ADKDAQDSREQTPLFLAAREGAVEVAQLLLELGAARGLRDQAGLAPGDVARQRSHWDLLTLLEGAGPTTQEARA------   1826

                         1847          1865                                                1917
AAC32288   1838   GPFPRARTVSVSVPPHGGGALPRCRTLSAGAGPRGGGACLQARTWSVDLAARGGGAYSHCRSLSGVGAGGGPTPRGRRFS   1917
AAC52630   1827   ----HARTTP------GGGSAPRCRTLSAGARPRGGGACLQARTWSVDLGARGGKVYARCRSRS--GSCGGPTTRGRRFS   1894

                                                                          Pest
AAC32288   1918   AGMRGPRPNPAIMRGRYGVAAGRGGRVSTDDWPCDWVALGACGSASNIPIPPPCLTPSPERGSPQLDCGPPALQEMPINQ   1997
AAC52630   1895   AGSRGR----------------RGARASQDDWPRDWVALEACGSACSAPIPPPSLTPSPERGSPQVAWGLPVHQEIPLNS   1958

AAC32288   1998   GGEGKK   2003
AAC52630   1959   VVRNLN   1964
```
